# The HSP70 Modulator MAL3-101 Inhibits Merkel Cell Carcinoma

**DOI:** 10.1371/journal.pone.0092041

**Published:** 2014-04-02

**Authors:** Christian Adam, Anne Baeurle, Jeffrey L. Brodsky, Peter Wipf, David Schrama, Jürgen Christian Becker, Roland Houben

**Affiliations:** 1 Department of Dermatology, University Hospital of Würzburg, Würzburg, Germany; 2 Departments of Chemistry and Biology, University of Pittsburgh, Pittsburgh, Pennsylvania, United States of America; 3 Division of General Dermatology, Medical University of Graz, Graz, Austria; Boston University Medical School, United States of America

## Abstract

Merkel Cell Carcinoma (MCC) is a rare and highly aggressive neuroendocrine skin cancer for which no effective treatment is available. MCC represents a human cancer with the best experimental evidence for a causal role of a polyoma virus. Large T antigens (LTA) encoded by polyoma viruses are oncoproteins, which are thought to require support of cellular heat shock protein 70 (HSP70) to exert their transforming activity. Here we evaluated the capability of MAL3-101, a synthetic HSP70 inhibitor, to limit proliferation and survival of various MCC cell lines. Remarkably, MAL3-101 treatment resulted in considerable apoptosis in 5 out of 7 MCC cell lines. While this effect was not associated with the viral status of the MCC cells, quantitative mRNA expression analysis of the known HSP70 isoforms revealed a significant correlation between MAL3-101 sensitivity and HSC70 expression, the most prominent isoform in all cell lines. Moreover, MAL3-101 also exhibited *in vivo* antitumor activity in an MCC xenograft model suggesting that this substance or related compounds are potential therapeutics for the treatment of MCC in the future.

## Introduction

Merkel Cell Carcinoma (MCC) is a highly aggressive neuroendocrine skin cancer, which primarily affects elderly or immunocompromised individuals [1], [2]. MCC is a rare disease, but its incidence is rapidly increasing [3], [4]. Treatment of primary tumors includes surgical resection and adjuvant radiotherapy [5], [6]. Therapeutic options for advanced disease are of limited efficacy with no proven benefit on overall survival [7]–[11]. MCC is associated in the majority of cases with Merkel cell polyoma virus (MCPyV). Indeed, MCC represents the human cancer with the best experimental evidence for a causal role of a polyoma virus, and expression of the T antigens by MCPyV is required for growth of MCC cells in cell culture and in xenografts [12], [13]. In particular, MCC cells depend on large T antigen (LTA) and its ability to interact with Retinoblastoma protein (Rb) [12]. It is believed that this interaction requires the activity of a cellular heat shock protein 70, or HSP70 [14].

Members of the HSP70 superfamily are highly expressed in many cancers [15], [16]. Notably, high HSP70 expression is associated with poor prognosis and resistance to chemotherapy while low HSP70 levels correlate with reduced tumorigenicity [15], [17]. The HSP70 superfamily is evolutionary highly conserved and consists of 17 isoforms [18]. Besides stress-inducible variants the family also includes the constitutively expressed HSC70 (HSPA8) [19]. HSP70 proteins are ATP-dependent molecular chaperones that regulate diverse cellular functions, including folding and assembly of newly synthesized as well as refolding of misfolded proteins, transport of proteins across intracellular membranes and maintenance of protein homeostasis within the cell [20]. Moreover, HSPs can interfere with cell death at different stages by blocking apoptosis in a caspase-dependent or independent manner [15], [21]. While the precise mechanisms by which HSP70 exerts its anti-apoptotic function are not yet fully understood, inactivation of HSP70 may hold great therapeutic value as HSP70-inactivating antisense oligonucleotides efficiently triggered cell death and cell cycle arrest in cancer cells [19], [22], [23].

MAL3-101 is a small molecule HSP70 inhibitor and exerts anti-proliferative and pro-apoptotic effects on cell lines derived from various cancers, including small cell lung carcinoma [24], [25]. MAL3-101 is a membrane permeable dihydropyrimidine analog that modulates the ATPase activity of HSP70 proteins and, in particular, inhibits the ATPase activity induced by simian virus 40 LTA, which interacts with HSP70 proteins via its J-domain [26]. LTAs promote G1/S cell cycle progression by inactivating proteins from the Rb family [27]–[29]. Notably, both the J-domain of LTA and HSC70-dependent ATP hydrolysis is required for Rb inactivation [30]–[32].

Although it has not yet been established whether binding of HSC70 by MCPyV LTA is required to support proliferation of MCC cells, it has been demonstrated that MCPyV LTA binds HSC70 via the J-domain, and that this interaction facilitates MCPyV replication [14]. As HSP70 proteins generally support growth and survival of tumor cells and may be particularly critical for MCPyV-transformed MCC cells, we evaluated the impact of MAL3-101 on MCC cell lines. These experiments revealed apoptosis induction *in vitro* as well as significant MCC tumor inhibition *in vivo* in a xenograft murine model. Strikingly, the efficiency of MAL3-101 correlated with HSC70 expression, but did not require the presence of MCPyV LTA in the analyzed cells.

## Material and Methods

### Ethics statement

The presented work was conducted according to the principles expressed in the Declaration of Helsinki. The generation and characterization of MCC cell lines was approved by the Institutional Review Board of University Hospital Würzburg (sequential study number 124/05). All the animal experiments were approved by the local authorities (government of Unterfranken; animal experiment request AZ: 55.2-2531.01-*59/06*) according to the legal requirements.

### Cell lines, cultures and reagents

The human MCPyV+ cell lines WaGa, BroLi, MKL-1 and MKL-2 as well as the MCPyV- MCC cell lines UISO, MCC13 and MCC26 have been described previously [13], [33]–[35]. In addition, this cell panel was complemented by the melanoma cell line FM88 [36], the T-cell line Jurkat [37] and HaCat keratinocytes [38], primary human keratinocytes and primary human fibroblasts. Primary human keratinocytes were obtained from foreskin and cultured as described [39]. Primary human fibroblasts were isolated from skin biopsies as described by Green et. al [40]. Recently, it was suggested that UISO might be not representative of Merkel cell carcinoma [41].

All MCC cell lines, as well as the FM88 and Jurkat line were grown in RPMI1640 (PAN Biotech, Aidenbach, Germany) supplemented with 10% (v/v) FCS (Biochrom AG, Berlin, Germany), 100 U/ml penicillin, and 100 μg/ml streptomycin (Sigma Aldrich, Munich, Germany), and maintained in a humidified atmosphere with 5% (v/v) CO_2_.

### Trypan-blue exclusion assay

Cell proliferation and viability under MAL3-101 treatment was measured using the trypan blue exclusion assay. After treatment, the cells from each group were stained with 0.4% trypan blue (Sigma Aldrich GmbH) and the number of dye-excluding (living) cells and positively blue stained (dead) cells was counted using a haemocytometer.

### Annexin V assay and cell cycle analysis

Apoptosis analysis was performed using the PE Annexin-V Apoptosis Detection Kit (BD Biosciences, Heidelberg, Germany) according to the manufacturer's instructions. Early apoptotic cells are identified as Annexin-V-positive and 7-AAD-negative, while late apoptotic cells are positive for both.

For cell cycle analysis, DNA staining was performed following overnight fixation with ethanol (90%). Cells were then pelleted and resuspended in PBS supplemented with 1% FCS, 0.05 mg/ml propidium iodide (Sigma Aldrich GmbH), and 0.1 mg/ml RNase A (Fermentas GmbH, St. Leon-Rot, Germany). After a one hour incubation at 37°C, cells were measured on a FACSCanto flow cytometer (BD Biosciences). Data were evaluated using FlowJo analysis software (Tree Star, Inc., Ashland, OR, USA).

### Real time polymerase chain reaction (PCR) for HSP70 isoforms

Relative expression levels of the HSP70 isoforms were determined by SybrGreen real time PCR applying the comparative ΔΔ−C_T_ method. Total cellular RNA was isolated using the RNAeasy kit (Qiagen, Hilden, Germany) with a subsequent DNaseI digestion step according to the manufacturer's instructions followed by cDNA synthesis with the Superscript II RT First Strand Kit (Invitrogen GmbH, Karlsruhe).

Real time PCR was conducted in the ABI 7500 Fast Real-Time PCR cycler (Applied Biosystems Inc., Foster City, CA, USA). The standard PCR reactions (20 μl) contained 1 μl cDNA and 10 μl 2× SybrGreen I Low Rox Mastermix (Eurogentec GmbH, Cologne, Germany) for detection. The thermal cycling conditions comprised an initial denaturation step at 95 °C for 10 min, followed by 40 cycles of three-step PCR including 15 sec at 95 °C, 60 sec at 60 °C and 30 sec at 95 °C. The C_T_ levels of the investigated HSP70 isoforms were normalized to GAPDH (Δ-C_T_ level). Δ-C_T_ was calculated as C_T HSP isoform, sample_ - C_T GAPDH, sample_.

Primer pairs for the different HSP70 isoforms were selected from NCI qPrimerDepot (Table 1) (http://primerdepot.nci.nih.gov/).

**Table 1:** Primer pairs for HSP70 isoform quantification by real-time PCR.

	primer sequence	
HSP70 isoform	Forward	Reverse
**HSPA1B**	5‘-TGAAGCAGCAAAGAGCTGAA-3‘	5‘-GTGGATTAGGGGCCTTTGTT-3‘
**HSPA2**	5‘-AAAGTTTGCTGATGATGGGG-3‘	5‘-TCGACAAGTGTCAGGAGGTG-3‘
**HSPA4**	5‘-TACCTGGCTTTTAGCTGCTG-3‘	5‘-CGCTAATGAGTATAGCGACCG-3‘
**HSPA5**	5‘-TGATTGTCTTTTGTCAGGGGT-3‘	5‘-CACAGTGGTGCCTACCAAGA-3‘
**HSPA6**	5‘-GATAAGTCAGCTGTGACTGTCAGG-3‘	5‘-TTATTTGAAGCAGAAGAGGATGAA-3‘
**HSPA7**	5‘-TTCCATGAAGTGGTTCACGA-3‘	5‘-TTGACGCTGGTGTCTTTGAG-3‘
**HSPA8**	5‘-TGGAAAACACCCACACAAGA-3‘	5‘-TCCTTCGTTATTGGAGCCAG-3‘
**HSPA9B**	5‘-ATTGAGCACGGGTCAACTTC-3‘	5‘-ATGGCACTTCAGAGGGTACG-3‘
**HSPA12A**	5‘-CAGGAATAACGCCTCTGTCC-3‘	5‘-CACCACGAGAAATGACTGCT-3‘
**HSPA14**	5‘-ATTCGAGAAGACCCTCCACA-3‘	5‘-GCAATGTGTCCAGAGCAAGA-3‘
**APG-1**	5‘-CATTTCCAATGGCTCGAGTT-3‘	5‘-CATTGCTGTCGCGAGAAGT-3‘
**STCH**	5‘-GAGCCAACATATTCTGGGGA-3‘	5‘-GGCTGAAATTGGCAGATACC-3‘
**HSPH1**	5‘-CGGCCATGAAATCTTTTGAA-3‘	5‘-AGACCATCGCCAATGAGTTC-3‘
**HYOU-1**	5‘-CTGCTCATTGAAAAGCCCA-3‘	5‘-CTGCAGATCCGGGGAGTAG-3‘

### Xenograft model, tumor induction and treatment protocols

Female NOD.CB17/JHliHsd-*Prkdc* ^scid^ mice were purchased from Harlan Laboratories GmbH (Eystrup, Germany) at the age of 4-6 weeks. All mice were housed under specific pathogen-free conditions according to the animal care guidelines in the Department of Dermatology of the University Hospital Würzburg (Germany).

Tumors were established by injection of 5×10^6^ WaGa cells (mixed 1∶2 with MatriGel, BD Biosciences) in a final volume of 100 μl subcutaneously into the lateral flank. The volume of the tumors was determined by measuring the diameter in two dimensions with a slide gauche and applying the following formula: V =  π/6 × a^2^ × b (a: length; b: width) twice weekly. When the tumors reached a volume of approximately 100 mm^3^, the mice were randomly divided into two groups (n = 6): i.e., the vehicle control group (mice injected intraperitoneally with 200 μl of PBS/20%DMSO every second day) and the MAL3-101 group (mice injected intraperitoneally with 40 mg/kg MAL3-101 every other day). The mice were closely monitored. On day 38, the experiment was terminated due to tumor size in the control group.

### Immunohistochemistry (IHC)

To measure the levels of HSP70 expression *in vitro* and *in vivo*, either cultured cells embedded in a bovine plasma/thrombin (5∶1) clot prior to formalin fixation, archived formalin-fixed and paraffin-embedded (FFPE) tumor samples from MCC patients, or FFPE samples from xenografted tumors were used. Antigen retrieval was achieved by incubation of the de-paraffinized sections with Dako Target Retrieval Solution (Dako, Hamburg, Germany), pH 6.0. For inactivation of endogenous peroxidases, slides were incubated with peroxidase blocking solution (Dako, Hamburg, Germany). Subsequently, sections were incubated overnight with the primary antibody (HSPA4/8, 1∶100, mouse monoclonal antibody, clon N27F3-4, Abnova GmbH, Heidelberg, Germany; Cleaved Caspase-3 (Asp175), 1∶1000, rabbit monoclonal antibody, Cell Signaling Technology, Inc., Danvers, MA, USA) in a humidified chamber at 4°C. For detection, the EnVision system-polymer HRP-labelled anti-rabbit or anti-mouse secondary antibodies (Dako) were applied followed by incubation with the Nova Red substrate kit (Linearis, Wertheim, Germany) according to the manufacturer's protocol. Finally, slides were counterstained with Mayer's hematoxylin (Dako).

### Statistical analysis

All quantitative assays were performed in triplicate. To evaluate the correlation between HSC70 expression levels and MAL3-101 sensitivity, relative gene expression levels were compared with the percentage of living cells after MAL3-101 treatment. Gaussian distribution of the data was confirmed by using the Shapiro-Wilk normality test. The correlation was determined using Spearman's correlation coefficient. Tumor volumes are expressed as means +/− SD. The related 2-way ANOVA was performed to determine significance of differences in tumor volume between vehicle and MAL3-101 treated animals. The statistical analysis was performed with GraphPad Prism 5.03 software (GraphPad Software, Inc. La Jolla, CA, USA). A value of p < 0.05 was considered significant.

## Results

### HSC70 is the most prominent HSP70 isoform in MCC cell lines

**Figure 1:**
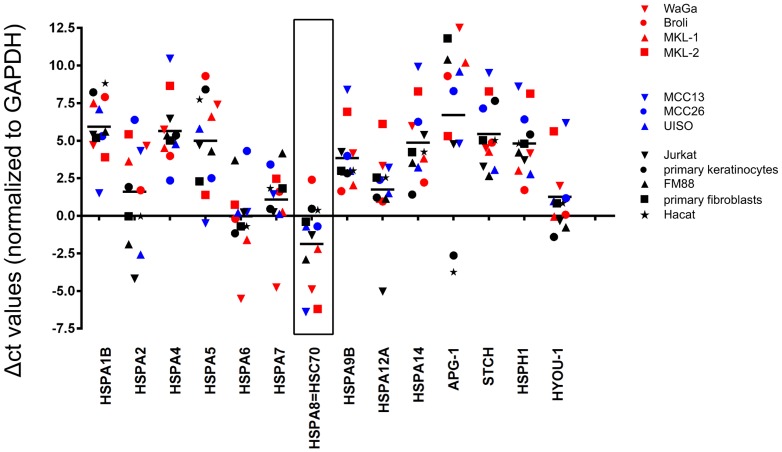
HSC70 (HSPA8) is the most prominent HSP70 isoform in MCC cell lines. Δ−C_T_ levels of the known HSP70 isoforms were determined applying SybrGreen real time PCR (red: MCPyV-positive; blue: MCPyV-negative; black: controls).

**Figure 2:**
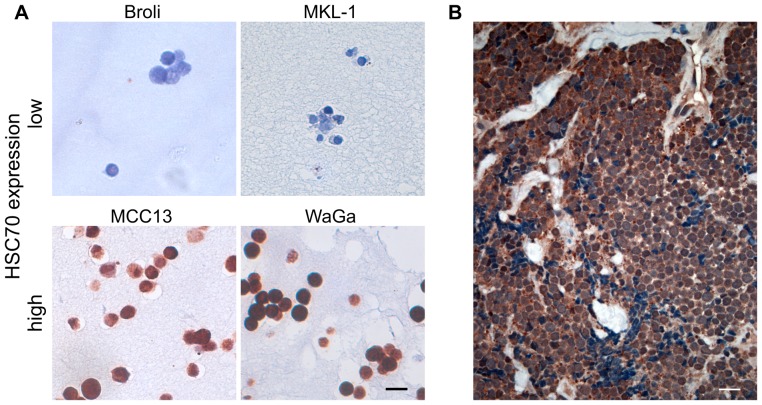
HSC70 protein expression *in vitro* and *in vivo*. HSC70 expression was assayed by immunohistochemistry applied to MCC cell lines embedded in a bovine plasma/thrombin clot prior to formalin fixation (A) and FFPE MCC metastasis of a patient (B). The depicted scale bar measures 20 μm.

Since HSP70s are frequently overexpressed in cancer cells, we determined mRNA expression levels of all known human HSP70 genes (excluding pseudo genes) in seven MCC cell lines using SybrGreen based quantitative PCR assays (Fig. 1). To this end, the mean expression of the isoforms HSPA1B, HSPA4, HSPA14, APG1, STCH and HYOU-1 was low (high Δ−C_T_), although expression varied widely among the different cell lines, in particular for HSPA2. For all other HSP70 isoforms, mRNA expression levels were high, i.e. equal to or above the mRNA expression of the housekeeping gene GAPDH. Notably, HSC70 was the most abundantly expressed mRNA in all MCC cell lines (low Δ−C_T_) and relative HSC70 transcript levels in the MCC cells were higher than those in all non-transformed control cells (HaCat, primary keratinocytes, primary fibroblasts). To confirm that HSC70 mRNA levels correlated with protein expression, we performed immunohistochemistry with cells of the respective MCC cell lines embedded in a bovine plasma/thrombin clot prior to formalin fixation. This analysis revealed a strong staining for HSC70 in the cell lines WaGa and MCC13 (relative mRNA expression compared to primary fibroblasts, the cell line with the lowest expression: 22.6 and 64.0, respectively, data not shown); however, under the same staining conditions, HSC70 was hardly detectable in the BroLi and MKL-1 cell lines (relative mRNA expression: 4.0 and 3.5, respectively, data not shown) (Fig. 2A). Moreover, we stained patient derived tumor tissues for HSC70 expression, demonstrating its presence in MCC cells *in situ* as well (Fig. 2B).

### The HSP70 inhibitor MAL3-101 induces apoptosis in MCC cell lines irrespective of their viral status

**Figure 3:**
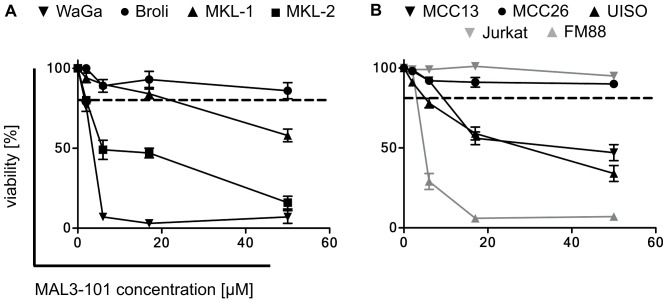
Heterogeneous MAL3-101 sensitivity of MCPyV-positive and MCPyV-negative tumor cell lines. Cell lines were seeded with 10000 cells in 96-well plates and incubated for 24 h before they were treated for 72 h with the indicated concentrations of MAL3-101. Cell viability was determined by the trypan blue exclusion assay (A, B). Given are mean values (+/- SD) of three independent experiments. FM88 is a melanoma cell line, Jurkat cells are derived from a T-cell leukemia, while all other cell lines have been established from primary or metastatic MCCs. The MCPyV status was determined by real time PCR and by immunohistochemistry for the MCPyV Large T antigen. MCPyV-positive cell lines are grouped in A and C, and MCPyV-negative in B and D. The dashed line indicates an arbitrary threshold (80% viability) allowing the discrimination between MAL3-101 sensitive and resistant cell lines.

**Figure 4:**
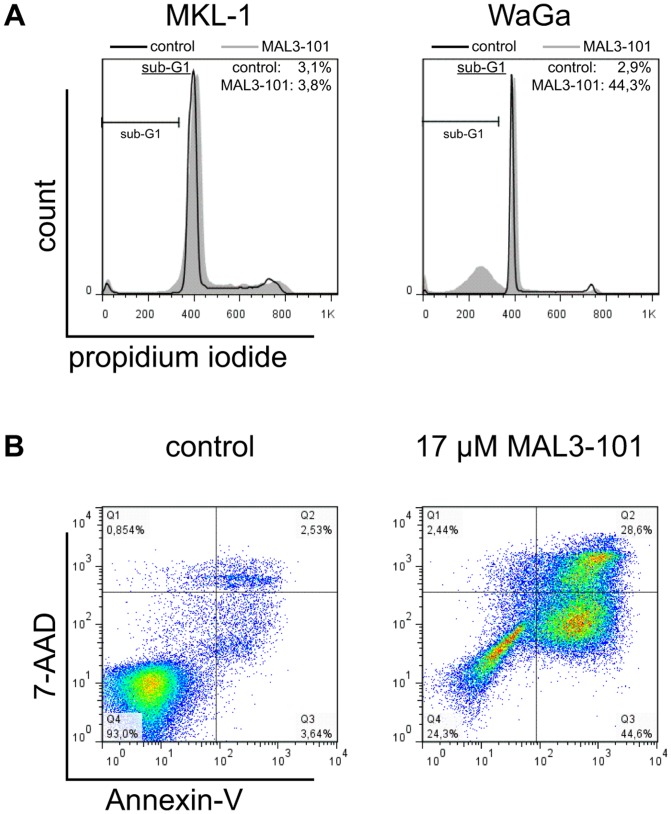
MAL3-101 induces apoptosis in cell lines with high HSC70 expression. (A) DNA staining of MAL3-101 treated WaGa cells (relative HSC70 mRNA level compared to primary fibroblasts: 22.6) reveals a strong increase in the sub-G1 population upon 20 h MAL3-101 treatment. In contrast, treatment of MKL-1 cells (relative HSC70 mRNA level compared to primary fibroblasts: 3.5) did not result in induction of a sub-G1 population. (B) Annexin-V/7AAD staining demonstrates increased early apoptosis (quadrant 3) as well as increased cell death (quadrant 4) in WaGa cells caused by MAL3-101 treatment (17 μM).

HSC70 is required for SV40-mediated cellular transformation through its interaction with the LTA [42]. Therefore HSC70 or other HSP70 isoforms may – in addition to other tumor-promoting abilities – be of particular relevance for MCPyV associated MCC by functioning with the MCPyV LTA. Consequently, we studied the impact of the HSP70 inhibitor MAL3-101 on MCPyV-positive (Fig. 3A) and MCPyV-negative tumor cell lines (Fig. 3B). The MCPyV-positive cohort included four MCC cell lines that all strictly require MCPyV LTA expression for maintenance. The MCPyV-negative cell lines investigated included three putative MCC cell lines, one melanoma cell line (FM88), and one T cell leukemia cell line (Jurkat). In either group, some cell lines responded very sensitively to MAL3-101 and demonstrated loss of viability, whereas others were hardly affected by the inhibitor (Fig. 3). These observations indicate that the presence of the polyoma virus is not critical for sensitivity towards MAL3-101, a notion that is in agreement with the effect of MAL3-101 in small cell lung cancer and myeloma [24], [25]. Nevertheless, five out of seven (72%) MCC cell lines responded to MAL3-101, suggesting that MCC can be targeted by HSC70 inhibition. The Annexin-V assay revealed a phosphatidylserine translocation in a large proportion of sensitive cells after treatment, and DNA degradation was evident (Fig. 4A and B). These data suggest that cell death induced by MAL3-101 is via an apoptotic pathway.

### Significant correlation of MAL3-101 sensitivity with HSC70 expression

**Figure 5:**
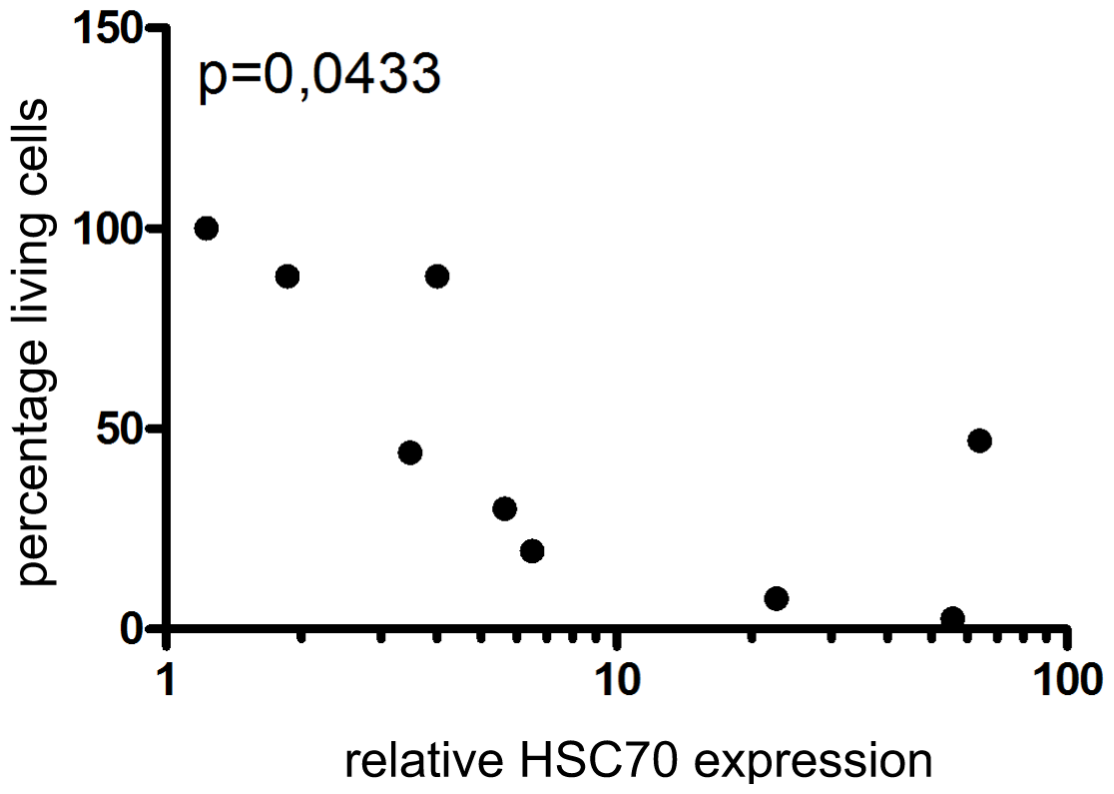
MAL3-101 sensitivity of MCC cell lines correlates with HSC70 expression levels. The relative HSC70 mRNA expression levels (with the lowest value arbitrarily set to 1) of the investigated cell lines were blotted against viability following 72 h of treatment with 17 μM MAL3-101. Gaussian distribution of the data was confirmed by using the Shapiro-Wilk normality test. Spearman's correlation coefficient as statistical test was applied.

Since the apoptotic response of tumor cells to MAL3-101 was not dependent on the presence of MCPyV, we tested whether MAL3-101 sensitivity would correlate with the level of HSP70 expression. Indeed, the viability of the treated MCC cell lines displayed a significant correlation with HSC70 mRNA expression (p = 0.0433, Spearman R = −0.7029) (Figure 5).

### MAL3-101 induces apoptosis in vivo and inhibits tumor growth

**Figure 6:**
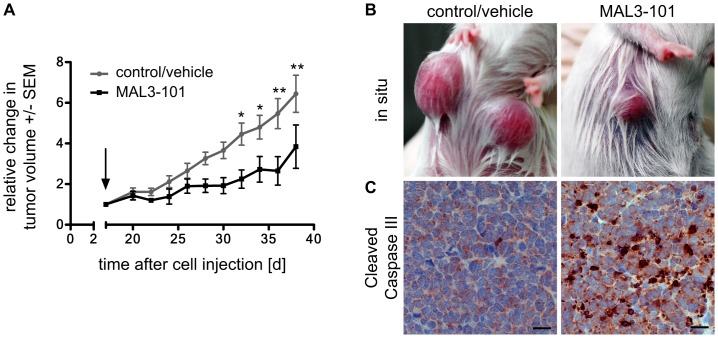
MAL3-101 treatment in an MCC xenotransplantation model demonstrated induction of apoptosis and reduced tumor growth. WaGa cells embedded in MatriGel were injected s.c. in NOD.CB17-*Prkdc^scid^*/NCrHsd mice. Intraperitoneal injection of MAL3-101 (40 mg/kg) was started (arrow) on day 17 when the tumor volume had reached approximately 100 mm^3^ and was repeated every second day. (A) Mean values (± SEM, N = 6) of the tumor volume are depicted in the graph. The p-value was calculated using the 2-way ANOVA statistical test. (B) Representative macroscopic photographs depict the respective tumors at day 38. (C) IHC for cleaved caspase III in FFPE tumors excised on day 38 indicating caspase III dependent apoptosis induction in the MAL3-101 treated group compared to control group. The depicted scale bar measures 20 μm.

To test whether MAL3-101 can affect MCC *in vivo*, we used a recently established xenotransplantation model [12]. Following subcutaneous injection of WaGa cells in NOD/Scid mice, animals were treated with the inhibitor once palpable tumors reached a size of approximately 100 mm^3^. Animals receiving 10 doses of 40 mg/kg MAL3-101 systemically over 21 days showed significantly reduced tumor growth compared to the vehicle control group (Fig. 6A and B). Tumor tissue was stained for cleaved caspase III, revealing that the MAL3-101 treated tumors showed a significantly higher presence of the cleaved form of caspase III compared to the control group. Thus, MAL3-101 also induces apoptosis in sensitive MCC cells *in vivo* (Figure 6C). Moreover, we could not detect any abnormalities or limitations in the animal behaviour or toxic side effects by MAL3-101 treatment suggesting that systemic delivery of MAL3-101 was well tolerated.

## Discussion

The five-year overall survival rate for patients with MCC is 40% and the relative survival rate (compared to an age- and sex-matched population) is 54% [39]. High mortality is largely due to the high propensity even of small primary MCCs to metastasize, and the resulting metastatic disease is treated by various combinations of chemotherapeutics [43]. However, although good initial response rates are achievable, the duration of these responses is generally short and it has not been conclusively demonstrated that any measure improves overall survival; thus, the median survival is less than a year [6], [11]. Therefore, there is a great medical need for alternative therapeutic approaches to treat MCC.

The discovery of a virus as a likely molecular cause for most MCCs will facilitate the rational design of targeted therapeutics. MCPyV was first described in 2008, followed by identification of the viral oncogenes, i.e. the early genes or T antigens, and the demonstration of an oncogene addiction of most MCPyV-positive MCC cell lines to these early genes [13]. Based on this notion and on the assumption that in analogy to the SV40 encoded LTA, MCPyV LTA may also critically depend on the HSP70 activity for interference of Rb-E2F interactions, we wanted to test if HSP70 inhibition would be detrimental for MCPyV-positive MCC cell lines.

HSP70 family members have been suggested as possible targets for therapy of different cancer entities apart from MCC [44]–[46] based on the following observations: HSP70 proteins (i) are overexpressed in cancer cells and/or are induced upon chemotherapy, (ii) possess several cytoprotective and anti-apoptotic functions [47], and (iii) have been demonstrated to be essential for cancer cell survival [19]. In the case of MCpyV-positive MCC, the essential role of HSP70 in the inactivation of the tumor suppressor protein Rb by polyoma LTA [48]–[50] suggests that HSP70 may be particularly crucial for this cancer type.

Here, we demonstrate that treatment with MAL3-101, a specific inhibitor of HSP70 proteins, induces apoptosis in sensitive MCC cells. Indeed, five out of seven MCC cell lines responded to MAL3-101 at low micromolar concentration. Although we cannot unequivocally conclude that survival of the MCC cells is affected by the compounds ability to inhibit HSP70, these results suggest that HSP70 might be a suitable target for therapy of some MCCs and in particular that MAL3-101 is a candidate for treatment of MCC. This notion is further substantiated by the effects of MAL3-101 in a preclinical mouse model for MCC. Specifically, we discovered that MAL3-101 is well tolerated by the mice and leads to significantly reduced growth of the xeno-transplanted MCC tumors. However, not all MCC cell lines were sensitive to the HSP70 inhibitor; conspicuously, the variable response of MCC cell lines to MAL3-101 did not correlate with the virus status of these cells; i.e. both MCPyV-positive and -negative MCC cell lines may be sensitive or resistant to MAL3-101. Instead, the sensitivity of the MCC cell lines towards HSP70 inhibitor appears to correlate with the expression level of HSC70 suggesting that a high expression of this protein results in increased sensitivity to HSP70 inhibition.

The observation that one of the MCPyV-positive MCC cell lines (BroLi) is almost completely insensitive to MAL3-101 may be due to MCPyV LT - at least in this cell line - lacking HSP70 dependency as we have previously demonstrated that this cell line is sensitive to MCPyV T antigen knock down [13]. Until recently, it has only been shown that MCPyV LT binds HSC70 and that an intact HSC70 binding site – which is present in MCPyV sT and LT – is required for T antigen driven viral replication [14]. Whether HSC70 activity is necessary for the ability of MCPyV to inactivate Rb and to support growth of MCPyV-positive MCC cells has not yet been demonstrated. It is worth noting that select functional differences between the MCPyV T antigens and their SV40 homologs have been noted [51]–[53].

It was recently suggested that all MCCs are MCPyV associated [54], putting into question whether the MCPyV-negative cell lines that were all established before the identification of MCPyV are really MCC cells. Indeed, these cells lack expression of many of the MCC markers, in particular of cytokeratin-20 [13]. However, the strongest response towards MAL3-101 among the MCC derived cell lines is observed in two of the virus-positive cell lines. Therefore, irrespective of the question of the identity of the MCPyV-negative MCC derived cell lines, our data suggest that inhibition of HSP70 by small molecule inhibitors such as MAL3-101 may represent a novel therapeutic strategy for the treatment of MCC either alone or in combination with other emerging therapeutic options [11], [55], [56].
